# Sex Steroid Receptors in Polycystic Ovary Syndrome and Endometriosis: Insights from Laboratory Studies to Clinical Trials

**DOI:** 10.3390/biomedicines10071705

**Published:** 2022-07-14

**Authors:** Fazilah Abdul Hamid, Muhammad Azrai Abu, Abdul Kadir Abdul Karim, Mohd Faizal Ahmad, Nor Haslinda Abd. Aziz, Datu Agasi Mohd Kamal, Mohd Helmy Mokhtar

**Affiliations:** 1 Department of Physiology, Faculty of Medicine, Universiti Kebangsaan Malaysia, Kuala Lumpur 56000, Malaysia; fazilah80@yahoo.com (F.A.H.); agasi.mk@ums.edu.my (D.A.M.K.); 2 Advance Reproductive Centre, Hospital Canselor Tunku Muhriz, Kuala Lumpur 56000, Malaysia; azraiabu1983@gmail.com (M.A.A.); abdulkadirabdulkarim@yahoo.com (A.K.A.K.); drmohdfaizal@ukm.edu.my (M.F.A.); 3 Department of Obstetrics and Gynaecology, Faculty of Medicine, Universiti Kebangsaan Malaysia, Kuala Lumpur 56000, Malaysia; norhaslinda.abdaziz@ppukm.ukm.edu.my; 4 Department of Biomedical Sciences, Faculty of Medicine and Health Sciences, Universiti Malaysia Sabah, Kota Kinabalu 88400, Malaysia

**Keywords:** sex steroid receptor, PCOS, endometriosis, folliculogenesis, prinaberel, clomiphene, vilaprisan

## Abstract

Polycystic ovary syndrome (PCOS) and endometriosis are reproductive disorders that may cause infertility. The pathology of both diseases has been suggested to be associated with sex steroid hormone receptors, including oestrogen receptors (ER), progesterone receptors (PRs) and androgen receptors (ARs). Therefore, with this review, we aim to provide an update on the available knowledge of these receptors and how their interactions contribute to the pathogenesis of PCOS and endometriosis. One of the main PCOS-related medical conditions is abnormal folliculogenesis, which is associated with the downregulation of ER and AR expression in the ovaries. In addition, metabolic disorders in PCOS are caused by dysregulation of sex steroid hormone receptor expression. Furthermore, endometriosis is related to the upregulation of ER and the downregulation of PR expression. These receptors may serve as therapeutic targets for the treatment of PCOS-related disorders and endometriosis, considering their pathophysiological roles. Receptor agonists may be applied to increase the expression of a specific receptor and treat endometriosis or metabolic disorders. In contrast, receptor antagonist functions to reduce receptor expression and can be used to treat endometriosis and induce ovulation. Understanding PCOS and the pathological roles of endometriosis sex steroid receptors is crucial for developing potential therapeutic strategies to treat infertility in both conditions. Therefore, research should be continued to fill the knowledge gap regarding the subject.

## 1. Introduction

Polycystic ovary syndrome (PCOS) is one of the most prevalent hormonal disorders among women of reproductive age [1]. This syndrome is a combination of endocrine, metabolic and reproductive disorders that cause irregular menstrual cycles, dyslipidaemia, excessive body weight, oxidative stress, hyperandrogenism and infertility [2,3]. PCOS affects 5–10% of reproductive-aged women, and 40% of those affected experience infertility, making it the primary cause of anovulatory infertility [2]. In clinical practice, three different diagnostic criteria have been proposed for PCOS diagnosis, including the National Institutes of Health Criteria, the Rotterdam Criteria for PCOS and the Androgen Excess Society Criteria [4]. However, the Rotterdam Criteria are the most widely used and acceptable criteria for assessing and managing PCOS. At least two of the following three signs must be present for a diagnosis to be made: loss of ovarian function (oligo-anovulation), excess clinical or biochemical androgen or ultrasound evidence of a polycystic ovary. 

Reports on the prevalence of PCOS vary in the literature due to its multisymptomatic nature; strict diagnostic criteria yield a prevalence of 6%, whereas inclusive diagnostic criteria result in a prevalence of up to 20% among reproductive-aged women [5,6]. Within the PCOS population, oligo-anovulation was found in 56.6% of PCOS women, whereas hyperandrogenaemia was found in at least 60% of women with PCOS [3,7]. Other characteristics of reproductive abnormalities following PCOS diagnosis include poor oocyte quality and endometrial receptivity [8]. In addition to reproductive abnormalities, the metabolic nature of PCOS may also increase the risk of other metabolic complications in women, such as hypertension and insulin resistance [9]. Considering its alarming prevalence as the most common endocrine and metabolic disorder in women of reproductive age, elucidating the molecular and cellular mechanisms underlying the pathophysiology of PCOS is of paramount importance.

Endometriosis is a chronic, benign inflammatory disease defined by the presence of dysfunctional extrauterine endometrial tissue. This disease is an oestrogen-dependent condition affecting up to 10% of reproductive-aged women and 35–50% of women with pelvic pain and infertility [10]. Due to its incapacitating symptoms, endometriosis can be a debilitating disease, with symptoms of chronic pelvic pain, dysmenorrhea and dyspareunia. Endometriosis has a complicated and multivariate aetiology, and its pathophysiology is described by a number of not-fully-defined theories [11]. 

The exact pathophysiology of PCOS and endometriosis is still not fully understood. However, research has explored the involvement of sex steroid hormone receptors, such as oestrogen receptors (ERs), progesterone receptors (PRs) and androgen receptors (ARs) in PCOS and endometriosis [12,13]. Furthermore, current studies on the structural malformation and associated malfunction of these receptors have triggered a new avenue for understanding PCOS and endometriosis aetiology. In this review, we will critically appraise and discuss recent advancements in understanding of the roles of sex steroid receptors in mediating the pathophysiology of PCOS and endometriosis.

## 2. Characteristics of Sex Steroid Receptors

### 2.1. Structure

Oestrogen receptors (ERs) were originally thought to exist only as nuclear receptors with two identified isoforms, namely ERα and ERβ [14]. Later findings revealed that oestrogen could also bind and activate the membrane-bound G-protein-coupled receptor, namely G-protein oestrogen receptor (GPER), acknowledging the existence of two classes of ER. [15] Nevertheless, GPER binds oestrogen with less binding affinity than the two other nuclear ERs. All three ERs are encoded by distinct genes located in different chromosomes. ERα is a 67 kDa protein comprising 595 amino acids from the ESR1 gene on chromosome 6. ERβ protein comprises 530 amino acids, with an approximate molecular weight of 59 kDa, with the gene encoding GPER located on chromosome 7 [16]. Various isoforms of each ER also exist due to selective splicing of transcripts. Three ERα isoforms, including ERα66, ERα36 and ERα46, have been identified. These isoforms differ in terms of the binding domain segments that are available to bind with DNA or transcription factors. The four identified isoforms for ERβ are ERβ2, ERβ3, ERβ4 and ERβ5, which all have variants in the C terminus and do not bind to oestrogen ligands [17]. A conformational change occurs upon binding oestradiol (E2) to ERα or ERβ in the cytoplasm, inducing dimerisation of the receptor. This complex is translocated to the nucleus, which binds to chromatin at the ERE sequences and alters the transcription of target genes by recruiting transcription factors [16]. Moreover, rapid responses to oestrogen mediated by GPER involve the mobilisation of various secondary messengers, such as cyclic adenosine monophosphate (cAMP) and calcium ions (Ca^2+^), or the activation of intracellular kinase pathways. These observations show that GPER can mediate these immediate effects to activate various signal transduction cascades, such as mitogen-activated protein kinase (MAPK), protein kinase C and phosphatidylinositol 3-kinase (PI3K) [18].

From a single gene located on chromosome 11, the human progesterone receptor (hPR) gene can be expressed as two distinct nuclear receptor isoforms: PR-A (94 kDa) and PR-B (116 kDa) [19]. The difference in the molecular weight is due to PR-A lacking 164 of the PR-B amino acids at the N terminal [20]. Progesterone binding to either PR-A or PR-B triggers homogeneous or heterogeneous dimerisation of the receptors. These dimerised complexes translocate into the nucleus and act as transcription factors that activate various subsets of genes. The variability of these combinations allows for the execution of distinct transcriptional functions and is considered a reflection of the progesterone effects [21].

In contrast, the androgen receptor (AR) is one of the 49 members of the steroid receptor family of ligand-activated transcription factors [22]. The AR gene, which is 90 KB in size, is located on the X chromosome and yields a 110 kDa nuclear receptor that can bind to only testosterone and dihydrotestosterone [23]. Other hormones, such as androstenedione (A4), dehydroepiandrosterone and dehydroepiandrosterone sulphate, act as prohormones that must be converted into testosterone before they can bind to AR and produce androgenic effects [24]. 

### 2.2. Distribution

**Figure 1:**
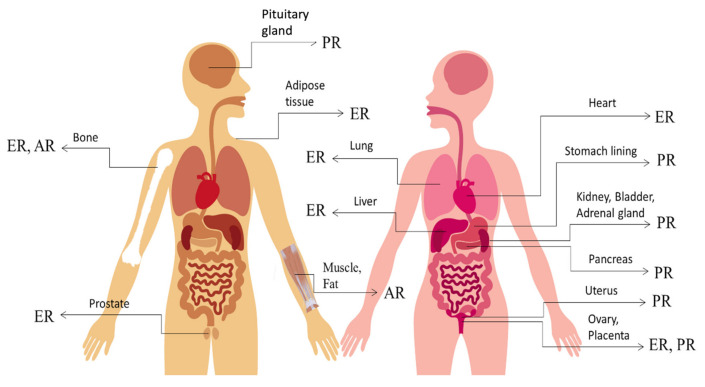
Distribution of sex steroid hormone receptors in males and females. ER: oestrogen receptor; PR: progesterone receptor; AR: androgen receptor.

Historically, oestrogen, progesterone and testosterone (androgen) have been known to exert effects exclusive to the development of the primary and secondary sexual organs. However, as research progresses and increased understanding is obtained, these hormones have been found to have wide-ranging effects as they travel throughout the body via the bloodstream. Figure 1 shows the distribution of sex steroid hormone receptors in males and females. Tissue-specific effects of sex hormones depend on the expression of their corresponding receptors in the tissues. Moreover, agonists and antagonists for the receptors are clinically relevant because they can exert different activation or inhibition effects and tissue-specific functions, depending on which tissue the receptors are expressed.

From radiolabelled-oestradiol binding assays to precise molecular techniques, such as PCR, detection of ER expression has allowed scientists to identify the target tissues where oestrogen has exerted its effects. Other than sex organ-related tissues, such as the prostate, ovaries and placenta, ER was also found to be expressed in the heart, bone, liver, lungs and adipose tissue [25]. To date, the role of ER in each target tissue has been substantially studied and discussed in a plethora of peer-reviewed publications. Hormone replacement therapy is one of the benefits derived from sex steroid receptor research. In addition, phytoestrogens and synthetic estrogenic compounds have been proven to be effective substitutes for impaired oestrogen production [26]. 

Similarly, the advancement of molecular biology has also allowed for the characterisation of PR expression profiles in many reproductive and non-reproductive tissues. Reproductive tissues expressing PR include the uterus, ovary and placenta, whereas non-reproductive tissues that express PR include the stomach lining, liver, pancreas, kidney, bladder, pituitary gland and adrenal gland [27]. 

Moreover, documented reports still restrict AR expression to tissues associated with sexual dimorphism articulation. These tissues include muscle, fat and bone tissues, where androgens can influence their growth and development to produce gender-specific characteristics. Additionally, studies on the role of AR in the nervous system suggest the hypothesis that postnatal activation of AR is responsible for potentiating specific aspects of male behaviour [28].

### 2.3. Physiological Functions

Sex steroid receptors are involved in regulating many essential functions of the body. For example, ER is involved in reproductive functions (follicular and endometrial development) and metabolic regulation (energy, glucose and lipid homeostasis), whereas PR is vital in reproduction (endometrial development), neuroprotection and metabolic regulation during pregnancy. In contrast, AR is involved in follicular and endometrial growth.

#### 2.3.1. Oestrogen Receptors

In follicular development, oestrogen receptors (ERs) are expressed specifically in the granulosa cells (GCs) and theca cells (TCs) of the ovarian follicles. TCs are stimulated by luteinising hormone (LH), resulting in the synthesis of androgens. Some of the androgens bind to AR, whereas others spread to GCs. In GCs, androgens are converted into oestrogens, mainly in the form of oestradiol (E2) under the stimulation of follicle-stimulating hormone (FSH). Increased levels of E2 bind to ERα, ERβ and GPER, which further increases the production of LH, leading to a surge of LH in GCs and TCs [29]. This condition stimulates the proliferation and differentiation of GCs and TCs, which is the final stage of follicle development, followed by the ovulation stage [30].

In endometrial development, ERα is present in the endometrial epithelial and stromal cells of the endometrium, ERβ is present in the glandular epithelial cells and GPER is localised in the plasma membrane of the endoplasmic reticulum. Increased levels of these receptors are observed during the proliferative stage under the stimulation of rapid endometrium growth by E2 [31]. As the primary mediator of oestrogenic action, the expression of ERα is higher than that of ERβ during the early proliferative stage [32]. Moreover, the expression of GPER is stimulated by E2 via ERα [33]. The binding of E2 to these receptors regulates the growth and proliferation of the endometrium. The expression of ERα, ERβ and GPER was found to peak in the late proliferative stage but decrease in the secretory phase, as oestrogen secretion decreased with the development of the corpus luteum and increased progesterone secretion [34].

In energy homeostasis, ERα, ERβ and GPER can be found in the cardiac [35] and skeletal muscles [36]. They are located in the nucleus, plasma membrane and mitochondria and act as nuclear receptors, transcription factors and signalling molecules for metabolic regulation, respectively [37]. Energy regulation in muscle tissues depends on the interaction between oestrogen and ER to modulate mitochondrial functions, such as ATP and reactive oxygen species (ROS) generation, antioxidant defences and mitochondrial biogenesis [38]. Translocation of the E2/ER complex into the nucleus occurs when oestrogen in the form of E2 binds to ERα and ERβ. Transcription of mitochondrial genes, Pgc-1α and NRFS occurs during interactions between the E2/ER complex and nuclear DNA [39,40]. These genes are involved in mitochondrial biogenesis, fatty acid utilisation and antioxidant defences [41]. E2 can also bind to GPER at the plasma membrane. This binding activates a series of intracellular signalling pathways, including PI3K, MAPK, extracellular signal-regulated kinases 1 and 2 (ERK1/2) and c-Jun-NH2-terminal protein kinase. These pathways are responsible for the transcription of genes encoding antioxidant enzymes, which further increases the antioxidant defences of the mitochondria [42]. As the primary ER isoform present in the mitochondria, ERβ binds to chaperone HSP27 and stabilises it [43]. Erβ is also involved in regulating mitochondrial DNA (mtDNA) transcription and replication and expressing respiratory-chain proteins by binding to the mtDNA [38]. In cardiac muscles, healthy mitochondrial function is essential to protect the heart from injury. GPER activation provides a protective effect on the heart after ischemia–reperfusion injury by inhibiting PTP opening via the ERK signalling pathway [44]. In addition, activation of ERβ protects the heart from trauma-induced haemorrhage by activating the mitochondrial biogenesis transcriptional cascade and inhibiting the mitochondria-mediated apoptotic pathway [45]. In skeletal muscles, normal mitochondrial homeostasis is crucial for muscle function and regeneration after injury. Activation of ERα by E2 regulates the expression of enzymes involved in fatty acid metabolism and increases the capacity of skeletal muscles to utilise fatty acids [46]. Activation of ERβ and GPER inhibits mitochondria-dependent apoptosis and mtDNA damage, respectively, caused by oxidative stress in the skeletal muscles [47].

In glucose homeostasis, ERα and ERβ are present in pancreatic β cells that produce, store and release insulin crucial for glucose homeostasis. E2 exerts its glucose homeostasis effect by binding to ERα and ERβ, increasing the biosynthesis and secretion, respectively, of insulin to control blood glucose levels [48]. In addition to insulin secretion, activation of ERα and ERβ by E2 can also promote the survival of pancreatic β cells under persistent cellular stresses, such as mitochondrial dysfunction, oxidative stress, cytokine-induced inflammation and glucolipotoxicity [49]. In lipid homeostasis, E2 binds to ERα and ERβ to modulate the central pathways involved in lipid metabolism, which includes lowering the levels of circulating lipids; reducing the expression of lipogenic and proinflammatory genes; and increasing the expression of lipid oxidation genes in the adipose tissues, liver and skeletal muscles [50]. As another ER class, GPER acts cooperatively with ERα and ERβ in mediating metabolic effects on glucose and lipid homeostasis through similar or distinct mechanistic pathways, respectively. Studies show that mice lacking either ERα, ERβ or GPER share similarities in terms of specific metabolic phenotypes, such as decreased insulin sensitivity, defective glucose and lipid homeostasis and increased adiposity [51]. 

Co-operative interaction between the two classes of receptors is essential to ensure that compensatory effects occur in the chronic absence of either one of the receptors. In glucose homeostasis, GPER activation by E2 stimulates the activation of the ERK signalling pathway, positively affecting insulin secretion. Furthermore, GPER activation simultaneously stimulates the PI3K pathway, inhibiting insulin secretion. Consequently, the two opposing pathways balance insulin secretion to maintain optimum blood glucose levels in the body [52]. In lipid homeostasis, activation of GPER helps co-ordinate lipid oxidation and prevent its accumulation in the liver and adipose tissues. This activation can be achieved by suppressing the expression of transcription factors responsible for lipogenesis, such as chSREBP and SREBP1, via the STAT3 signalling pathway. This phenomenon reduces lipotoxicity and has an antiapoptotic effect on adipocytes [53]. Lipid accumulation can also be prevented by promoting the differentiation of preadipocytes into adipocytes, as shown by the upregulation of GPER expression during the process. GPER activation prevents cell cycle arrest of preadipocytes by increasing the expression of cell cycle regulating factors, such as cyclin D, CDK4 and CDK6. Increased expressions of these factors then induces adipocyte differentiation [54].

#### 2.3.2. Progesterone Receptors

Progesterone receptor (PR) plays a more critical role in female reproduction than ER, especially in endometrial development for pregnancy. Before embryo implantation, PR-A and PR-B are present in the endometrial luminal epithelium during the proliferative phase. They both increase in accordance with the rising level of progesterone and decreasing oestrogen. During the ovulation phase, PR-A decreases, whereas PR-B remains constant, indicating the role of PRB in glandular secretion [55]. Progesterone binding to PR-B triggers the production of prostaglandins and cytokines attributed to the inflammatory-like characteristics of ovulation [49]. Following ovulation, fertilisation and progressive development of the zygote into activated blastocyst occur. Simultaneously, the corpus luteum is formed, and ovarian steroid hormone biosynthesis shifts from oestrogen to progesterone production. Moreover, the predominant PR-A in the endometrial stromal cells binds to progesterone and stimulates the proliferation of the stromal cells. Activation of PR-A by progesterone also suppresses the oestrogen-induced proliferation of endometrial epithelial cells, changing it into a differentiated state for embryo attachment and invasion. 

The underlying fibroblastic stromal cells undergo proliferation and differentiation into epithelioid decidual cells, a process mediated by PR-A, after embryo attachment [56]. Decidual cells provide an immunotolerant microenvironment for the implanted embryo, protect it from physiological stressors and prevent excessive embryonic invasion into the uterine compartment [57]. Progesterone-driven decidualisation depends on the expression ratio of PR-A and PR-B. A ratio favouring PR-B mediates the anti-inflammatory effects of progesterone based on the influx of uterine natural killer cells (uNKCs). uNKC is essential to promote an immunosuppressive milieu for the invading hemiallogeneic conceptus. These cells also promote the remodelling of spiral arterioles to facilitate endovascular trophoblast invasion, which ultimately forms the endotheliochorial placenta [58]. Conversely, a ratio favouring PR-A mediates the proinflammatory effects of progesterone. This phenomenon occurs when uNKC prevents excessive trophectodermal invasion into the uterine compartment by triggering apoptosis of the advancing trophoblastic cellular front of invading conceptus [59]. Progesterone-dependent uterine receptivity and decidualisation represent a critical step in ensuring pregnancy success. Therefore, as mediators of this process, maintaining the regular expression of PR-A and PR-B isoforms is important. The absence of either one would lead to infertility or other complications, such as miscarriage [60].

PR-A and PR-B can also be localised in different parts of the brain, including the hypothalamus, hippocampus and cortex. PR-A and PR-B activations by progesterone mediate neuroprotection effects on the brain via activation of the MAPK, ERK and Akt signalling pathways [61]. These neuroprotective effects include myelination, glial cell functions, inflammation and neurogenesis [62]. Progesterone binding to PR-A and PR-B induces myelin production on glial cells and helps slow the progression of Alzheimer’s disease [63]. Progesterone helps to correct and maintain neuronal homeostasis after traumatic brain injury through PR-A and PR-B [64]. Moreover, progesterone may protect against cerebral oedema and secondary neuronal death caused by traumatic brain injury. Furthermore, activation of PR-A and PR-B may inhibit the inflammatory cytokines IL-β and TNF-α in the frontal cortex of the traumatic brain injury, preventing cerebral oedema by stabilising the blood–brain barrier and inhibiting water, ions and inflammatory molecules from crossing the blood–brain barrier [65]. In addition, progesterone-driven activation of PR-A and PR-B can upregulate the expression of brain-derived neurotrophic factor, a neurotrophin in the central nervous system that functions to reduce mitochondrial dysfunction in the brain, offering another layer of protection against brain injury [61]. 

As the body adapts to pregnancy, progesterone and PR mediate various metabolic regulations to ensure sufficient nutrient supply for maternal and foetal needs. Thus, PR can be found in the pancreatic β-cells, adipose tissue, liver and skeletal muscles. Progesterone can regulate carbohydrate metabolism through PR by inducing hyperinsulinemia on pancreatic islets while promoting glycogen storage in the liver. PR activation in glucose metabolism promotes the transcription of gluconeogenic genes and increases blood glucose levels under insulin-resistant conditions [66]. Through the hepatic PR, progesterone also activates glycogen phosphorylase, an enzyme involved in glycogen metabolism, increasing blood glucose levels [46]. Several pregnancy studies have reported that high progesterone levels and PR expression correlate with incidents of gestational diabetes characterised by glucose intolerance due to inadequate insulin supply [66,67]. Another study showed that PR knockout female mice have high glucose tolerance and low fasting blood sugar due to increased pancreatic β-cell mass and proliferation [68]. This finding suggests that PR expression and progesterone have adverse effects on insulin production. 

In contrast, progesterone exerts a catabolic effect on lipid metabolism through PR by inhibiting lipolysis and promoting lipid storage in the adipose tissue and skeletal muscles. This effect is demonstrated in PR-expressing pregnant rat adipocytes, wherein lipolysis is inhibited [69]. The actions of progesterone and PR on metabolism during pregnancy are well-studied. However, the exact involvement of each isoform PR-A and PR-B in these regulations and the interaction between the two isoforms remain unclear.

#### 2.3.3. Androgen Receptor

Despite being a male sex hormone, androgen and androgen receptors (ARs) play an equally important role in female reproduction, especially in follicle and uterine development. AR expression can be detected in the ovary [70], fallopian tubes [71] and endometrium [72] throughout the menstrual cycle, which is consistent with the role of androgen in modulating the follicle cycle and uterine development. Furthermore, an experiment involving the generation of a transgenic AR reporter mouse, which expresses a luciferase reporter gene under the control of activated endogenous AR, showed high AR activity in the uterus and ovaries, proving the direct action of androgen in these tissues [73]. TCs produce androgens under the stimulation of LH during the early stage of the menstrual cycle. Part of the androgen binds to AR in the TCs, whereas some parts spread to GCs to be converted into oestrogen. The remaining unconverted androgen in the GCs binds to AR in the GCs. AR activation in the TCs and GCs stimulates the proliferation and differentiation of the cells into mature follicles [29]. Evidence has shown intense AR expression in TCs and GCs during the early/mid proliferative stage [70]. AR detection in primate ovaries is most abundant in immature follicles [74]. ARs also demonstrate FSH interactions during early follicular development, as proven by the increased AR expression by FSH in primary follicles. A positive correlation was also observed between AR expression and FSHR mRNA levels in GCs isolated from normal, androgen or FSH-treated primates [75]. AR expression in the ovaries decreases as the follicles mature during the later stage of follicular development [76]. 

The highest levels of AR expression are observed in the endometrial stromal cells within the upper functional layer of cells during the oestrogen-dominated proliferative stage of uterine development. AR was also found to be present in the basal layer of the stromal cells throughout the cycle [72]. This finding indicates the involvement of androgen in stimulating the proliferation of endometrial stromal cells through AR activation. As the process progresses, AR expression is upregulated in the glandular epithelium during the mid/late secretory phase when progesterone decreases due to the demise of the corpus luteum [72]. This finding, again, indicates the involvement of AR in the maintenance of the endometrium.

## 3. Sex Steroid Receptors in PCOS and Endometriosis

**Figure 2:**
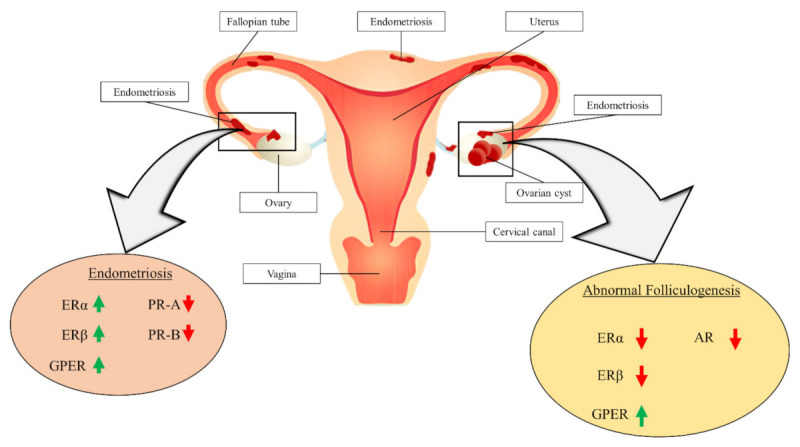
Association of receptor expression changes with the pathogenesis of abnormal folliculogenesis and endometriosis. ER: oestrogen receptor; PR: progesterone receptor; AR: androgen receptor; GPER: G-protein oestrogen receptor.

PCOS patients may be associated with the conditions of abnormal folliculogenesis, metabolic disorders and anovulation [77,78,79]. These conditions are related to changes in the expression of sex steroid receptors. Figure 2 demonstrates the association of receptor expression changes with abnormal folliculogenesis and endometriosis pathogenesis.

### 3.1. Abnormal Folliculogenesis in PCOS

Abnormal folliculogenesis refers to the failure of the ovarian follicle to mature into a functional ovum. The remaining undegenerated cystic follicle after ovulation failure leads to cysts in the ovary [80]. 

Disruption of ER expression plays a significant role in ovulation dysfunction associated with PCOS. In vitro studies showed that mice with altered ERα genes developed a PCOS-like phenotype, which is characterised by high circulating LH, haemorrhagic ovaries and cystic, non-ovulating follicles [81]. Similarly, another in vitro study comparing the mRNA and protein levels of ERα and ERβ in GCs and TCs between normal and polycystic ovaries demonstrated that ER expression is higher in GCs than in TCs for polycystic ovaries. In contrast, ERβ expression remained the same in GCs and TCs for both ovaries [82]. This finding indicates that impaired follicle development and anovulation are more related to the aberrant expression of ERα than ERβ. Moreover, ER knockout rats that fail to express functional ERα have polycystic ovaries, ovulation defects and uterine dysplasia. They also demonstrate prominent abnormalities in female genital development, fertility and response to E2 [83]. A human case study reported a female patient with a homozygous mutation in the ESR1 gene responsible for ERα expression with a small uterus and ovarian cysts [84]. Surprisingly, another in vitro study showed that abnormal expression of ERβ in mouse ovaries can also cause follicular development failure [85]. In addition to abnormal follicular development, ERβ knockout mice are also observed to have decreased ovulation ability [86]. Furthermore, a study showed that ERβ knockout mice have early atretic follicles and few corpora lutea, despite their preserved reproductive ability [87]. 

On the contrary, another publication reported that ERβ knockout mice display poor fertility due to low ovulation frequency, few pregnancies and reduced preovulatory follicle differentiation, all of which are attributed to weak response to FSH [88]. In addition to ER, another oestrogen-binding receptor, GPER, plays a significant role in the abnormal folliculogenesis associated with PCOS. High levels of GPER expression are detected in the cumulus GCs of PCOS patients, inhibiting oocyte maturation [89]. In contrast, an in vitro study showed that the growth of carp (Cyprinus carpio) oocytes during the final stage is significantly decreased when incubated with GPER agonist G-1 [90]. Moreover, research involving GPER small interfering RNA knockdown mice indicates that GPER has exerts a decisive inhibitory action on primordial follicles, proving its role in obstructing oocyte maturation [63]. 

Most women diagnosed with PCOS present with hyperandrogenism; thus, disruption in AR expression is also implicated in abnormal folliculogenesis and anovulation [91]. AR knockout mice exhibit subfertility [92]. Subfertility in mice is associated with a lack of androgen activity and low expression of AR in the GCs, which leads to a prolonged oestrous cycle and an increased number of preantral and atretic follicles with decreased corpora lutea and ovulation rates [92]. In a study on the expression of AR in luteinised GCs from 169 Chinese women (106 PCOS and 63 normal) aged between 20 and 35 years, qRT-PCR analysis revealed that AR mRNA expression in GCs of PCOS patients was significantly lower compared with normal women (*p*-value < 0.001). Furthermore, the levels of AR expression are low in PCOS patients with a significant number of antral follicles [12]. However, an inconsistency regarding the influence of different AR expression levels on PCOS abnormal folliculogenesis is observed. For instance, another in vivo study reported that AR is highly expressed in PCOS animal models due to induction by androgen, suggesting an association between PCOS and hyperandrogenism [89]. To explain this phenomenon, the authors of [12] hypothesised that AR expression might be increased by short androgen stimulation in vivo and in vitro. Afterwards, the expression of AR might be inhibited due to the increased activity of AR in a chronic hyperandrogenism environment of PCOS. This condition inhibits the normal development of the follicles, as AR expression decreases during folliculogenesis, leading to the formation of numerous large antral follicles in the ovaries of PCOS patients. Nonetheless, abnormal folliculogenesis in PCOS is associated with the abnormal expression of AR, regardless of overexpression or underexpression.

### 3.2. Metabolic Disorders in PCOS

Metabolic disorders commonly experienced by PCOS patients include obesity, diabetes mellitus, cardiovascular disease and hypertension. The pathophysiology of these conditions is often associated with disruption of the normal expression of sex steroid receptors. For example, abnormal expression of ER and GPER is associated with obesity, diabetes mellitus and cardiovascular disease. Previous discussions indicated that ER and GPER are responsible for lipid homeostasis; thus, changes in their expression affect the lipid metabolism of the body. Excess lipid accumulation increases body weight, leading to obesity. Reports have also indicated that mice lacking ERα, ERβ and GPER have increased body weight and visceral adiposity compared to wild-type mice with intact ERα and ERβ [93]. Increased adiposity is also observed in ER and GPER knockout mice. Results revealed an overall increase in lipid content in the body of ER and GPER knockout mice, with increased lipid deposition in the subcutaneous and visceral fat depots. Weight gain in the mice was also correlated with a significant increase in circulating cholesterol levels, triglycerides and low-density lipoproteins (LDLs) [50]. This phenomenon suggests that ER and GPER expression disruption impedes lipid metabolism, leading to obesity.

Similarly, impaired glucose regulation is also caused by abnormal expressions of ER and GPER. Deletion of ERα, ERβ and GPER in mice increases the severity of insulin-deficient diabetes compared with wild-type mice [94]. Moreover, impaired glucose tolerance, hyperglycaemia, loss of pancreatic β-cells and decreased pancreatic insulin levels can be detected in mice without ERα, ERβ and GPER [95]. This condition indicates that normal blood glucose levels cannot be maintained without the expression of ER and GPER, thus increasing the susceptibility of individuals to diabetes mellitus. Importantly, ER disruption can also cause cardiovascular diseases due to mitochondrial alterations in the cardiomyocytes. Quantitative proteomic analysis of cardiac mitochondria from an ERα knockout rat showed a significant reduction in mitochondrial proteins involved in electron transport chain (ETC), oxidative phosphorylation, regulation of oxidative stress and apoptosis, and lipid and carbohydrate metabolism [96]. Consequently, the activity of ETC complexes and the capacity for calcium handling decrease, and mitochondrial membrane lipid peroxidation in the cardiac muscles increases [97]. Moreover, ATP synthesis is driven by complex 1 and decreases as stress-induced ROS production increases in the cardiac mitochondria [98]. Cardiac mitochondrial biogenesis becomes obstructed as oxidative stress increases. This condition results in the death of cardiomyocytes, leading to myocardial infarction [99]. In addition to the lack of ERα expression, the inactivation of GPER may also cause mitochondrial alterations in cardiomyocytes through the downregulation of PGC-1α, which is the gene responsible for mitochondrial biogenesis. This condition is associated with concentric cardiac hypertrophy in menopausal women [100]. 

In addition to ER, abnormal expressions of AR are associated with obesity and diabetes mellitus in PCOS. Hyperandrogenism is one of the phenotypes associated with PCOS [91]. Thus, increased androgen activity mediated through elevated AR expression negatively affects lipid and carbohydrate metabolism. A study reported that ewes treated with androgen displayed several metabolic dysregulations, such as abolishing increments in visceral fat, pronounced adipocyte hypertrophy, fatty liver and hyperlipidaemia [101]. Similarly, metabolic abnormalities, including constraints on adipocyte maturation, diminished adipocyte release of adiponectin, increased intra-abdominal fat storage and hyperlipidaemia, are observed in a normal-weight PCOS patients with elevated androgen and AR expression [102]. All these abnormalities may lead to obesity. Increased AR expression in PCOS also causes dysregulation in hepatic glycogen in the absence of insulin resistance, leading to hyperglycaemia and diabetes mellitus [103]. Furthermore, hypertension is positively correlated with AR expression. Reports have indicated that androgen administration to female rats for three months invokes multiple cardiometabolic features, such as increased blood pressure, food intake, adiposity and renal injury [104]. The blood pressure of rats treated with androgen was found to be higher than that of untreated rats [105]. A study proved that the activation of the intrarenal renin-angiotensin system induces blood pressure elevation in PCOS, which is mediated by androgen via AR [106]. Another study reported that the blockade of AR reduces blood pressure and decreases renal angiotensin type 1 receptor mRNA expression in female PCOS rats [107]. All these findings confirm the pathophysiological role of AR in PCOS-linked hypertension.

### 3.3. Endometriosis

Endometriosis is a benign gynaecological disease whereby endometrial tissue is dispersed outside the uterus on the peritoneum, ovaries, bladder, rectovaginal septum or intestines. This disease may cause pain due to altered pelvic structure and extensive adhesions, leading to infertility in severe cases. Common symptoms of endometriosis in pelvic pain, dyspareunia and dysmenorrhoea [108].

One of the factors leading to endometriosis is the accumulation of oestrogen and activation of ER and GPER. Oestrogen accumulation occurs in the endometriotic tissues due to the high expression of aromatase and steroidogenic acute regulatory protein, the two enzymes responsible for the conversion of cholesterol into E2 [109]. The binding of excess E2 to overexpressed ER and GPER stimulates the proliferation of endometriosis-like lesions [85]. An experiment using ERα and ERβ knockout mice models to induce the growth of endometriosis-like lesions suggested that ERα and ERβ are associated with disease progression [110]. Similarly, increased GPER expression in women with endometriosis suggests the involvement of GPER in the disease [111]. Overexpression of ERα is associated with cell adhesion, proliferation and neoangiogenesis, which supports the growth of endometriosis-like lesions [110]. Elevated expression of ERβ is related to the inhibition of TNF-induced apoptosis and the increment of IL-1β secretion, which contributes to cell survival, proliferation, invasion and adhesion of immortalised human endometrial cells. Additionally, overexpression of ERβ leads to endometriosis-associated infertility because it inhibits the decidualisation response in the stromal cells of the endometrium [112]. In contrast, overexpression of GPER in the secretory endometrial epithelium is associated with the continued proliferation of shed endometrial cells in the peritoneal cavity [113]. A study by [114] detected a significant increase in the expression levels of ERα and ERβ in the endometriotic lesion in comparison with normal endometria. Another study revealed that the expression of ERβ mRNA increased by 34-fold in human endometriotic tissues compared with normal endometria [115]. Another study reported that the expression of ERβ mRNA increases by 34-fold in human endometriotic tissues compared with normal endometria [115]. Abnormally elevated GPER expression is observed in the eutopic and ectopic endometrium of women with endometriosis [113]. Additionally, analysis of macrophages obtained from peritoneal fluid of women with endometriosis shows a positive correlation between the expression of Erβ and proinflammatory cytokines in women both with and without endometriosis, whereas the expression of ERα positively correlates with selective cytokine production in women with endometriosis only [116]. 

In addition to ER and GPER, the dysregulation of PR expression is also related to endometriosis [117]. Progesterone resistance is hypothesised to induce the initiation of endometriosis development, as several studies have reported impaired progesterone action in the endometrium [117]. Evidence suggests that PR-B expression is either absent or high, whereas PR-A expression is notably downregulated in the superficial peritoneal lesions and ovarian endometriomas [118]. PR-A is the predominant PR isoform because PR-B deficiency is prominent in lesions [117]. However, in deep rectal lesions, PR-A and PR-B expression can be detected in the glands and stroma, albeit at significantly lower levels than in normal endometria [119]. PR-mediated progesterone signalling exerts antimitogenic effects on the endometrial epithelial cells, which is essential for the mitigation of trophic oestrogen effects on normal endometrium and ectopic implants of endometriosis [56]. However, the absence or underexpression of PR-A and PR-B disrupts normal progesterone antimitogenic signalling, resulting in the development of endometriosis.

## 4. Potential Therapeutic Routes

**Figure 3:**
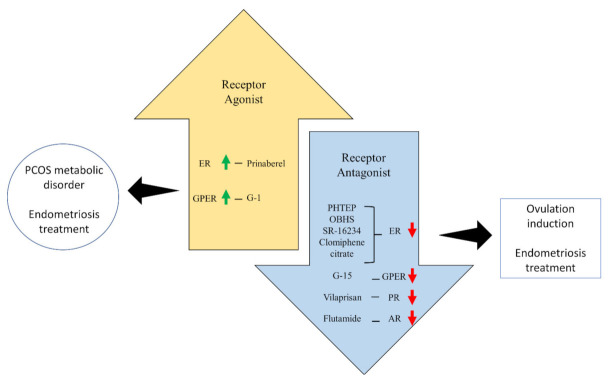
Summary of potential treatment routes for PCOS-related disorders. ER: oestrogen receptor; PR: progesterone receptor; AR: androgen receptor; GPER: G-protein oestrogen receptor; OBHS: oxabicycloheptene sulfonate.

One of the viable treatments for medical conditions experienced female patients with PCOS is the administration of selective receptor modulators, which control the activity of sex steroid receptors. The modulators can act as either agonists or antagonists to a specific receptor. A summary of potential treatment routes for PCOS-related disorders is shown in Figure 3.

### 4.1. Receptor Agonist

A receptor agonist is a receptor ligand that activates a biological response upon binding to a specific receptor.

Prinaberel (ERB-041) is an example of an ER agonist; it functions to restore the expression of ERβ. A study showed its effectiveness not only in restoring or augmenting ERβ expression in murine squamous cell carcinoma and human carcinoma cells but also in reducing proliferation and inducing differentiation and apoptosis in skin carcinogenesis [120]. The capability of prinaberel to reduce the proliferation of cancerous cells suggests its suitability as a therapeutic agent for endometriosis. Moreover, prinaberel has anti-inflammatory properties, which it exerts by suppressing the NFκB proinflammatory signalling pathway, which is responsible for the transcription of various proinflammatory genes, such as inducible nitric oxide synthase (iNOS), p65 protein, cyclooxygenase-2 (COX-2), IL-6 and IL-1β [120]. Furthermore, a study showed significant inhibitions of iNOS production in peritoneal macrophages of endometriosis following treatment with prinaberel [63]. In another study using a mouse endometriosis model to evaluate the efficacy of prinaberel in treating endometriosis, endometriotic lesions were generally regressed in 40–75% of the mice following prinaberel treatment [87]. This finding proves the anti-inflammatory benefits of prinaberel and its effectiveness in reducing lesions associated with endometriosis. However, several adverse events, such as headache, nausea, bronchitis and infection, were reported following prinaberel treatment [121]. Therefore, using prinaberel requires caution.

G-1 is an example of a selective receptor agonist for GPER, functioning to activate GPER expression. Treatment of an obese mouse model with increased adiposity using a G-1 agonist has been shown to attenuate overall weight gain and lipid content in mice. Furthermore, a significant reduction in the mass of multiple fat depots and increased energy expenditure in the brown adipose tissue without any adverse effect on bone mineral density and content or lean mass following G-1 treatment are observed [122]. Moreover, G-1 therapy improved glucose regulation in ovariectomised mice, as demonstrated by low levels of fasting glucose and insulin, increased insulin sensitivity, improved glucose tolerance, and reduced circulating proinflammatory cytokines and hormones (leptin and resistin). Low fasting glucose and insulin levels prove the beneficial effects of G-1 agonists on glucose regulation. Furthermore, this finding implies the direct role of G-1 in the regulation of glucose production and uptake in the liver and skeletal muscles, respectively [123]. Therefore, this phenomenon indicates that G-1 agonist may be a suitable therapeutic candidate to treat GPER-induced obesity and reduce lipid deposition or even alleviate chronic diseases related to obesity, such as diabetes mellitus.

### 4.2. Receptor Antagonist

A receptor antagonist is a receptor ligand that blocks a biological response upon binding to a specific receptor. For example, different antagonists for the sex hormone receptors, namely ER, PR and AR, have been developed to treat medical conditions caused by receptor dysregulation. 

PHTPP, oxabicycloheptene sulfonate (OBHS) and SR-16234 are examples of effective ER antagonists for endometriosis treatment. PHTPP exhibits antagonistic activity against ERβ. Additionally, PHTPP reduces ERβ expression, which enables the suppression of TNF activity, reducing the onset of endometriosis. The treatment of surgically-induced endometriosis lesions in mice with PHTPP was reported to successfully suppress ectopic lesion growth [112]. A significant reduction in endometriotic lesion size was also observed in a baboon endometriosis model following PHTPP treatment [124]. In contrast, OBHS exerts antagonistic effects on Erα, resulting in potent antiestrogenic and anti-inflammatory impacts on endometriotic lesions by regulating the level of ERα expression. Moreover, OBHS has been proven effective in reducing inflammation at the site of endometriotic lesions in a suture endometriosis mouse model, as well as on in vitro primary human endometriotic stromal cells [125]. SR-16234 exhibits ERα antagonistic activity coupled with a partially weak ERβ agonist activity. SR-16234 can suppress the expression of genes associated with inflammation in endometriosis-like lesions, such as TNF-α and IL-1β, preventing the growth and proliferation of endometriotic tissues. A study also demonstrated the effectiveness of SR-16234 in suppressing the growth of endometriosis-like lesions in a murine model of endometriosis. The results show that treatment SR-16234 significantly reduced the level of proinflammatory cytokines, namely TNF-α and IL-1β, in the endometriosis-like lesions [126]. 

Clomiphene citrate, which acts as a receptor antagonist for ER, is usually used for ovulation induction. Clomiphene citrate has two isomers, namely zuclomiphene and enclomiphene, which compete for receptor-binding sites with endogenous oestrogen. Zuclomiphene can induce ovulation and has a longer half-life than enclomiphene. The mechanism of action of clomiphene citrate involves inhibition of negative oestrogen feedback to the hypothalamus and pituitary gland by blocking ER. In addition, increased secretion of gonadotropins from the anterior pituitary can stimulate follicular development and ovulation [127]. Treatment of PCOS patients with a 50–150 mg daily oral dose of clomiphene for five days beginning on day 3–5 after automatic withdrawal bleeding significantly reduced ovulation time by an average of 30 days and simultaneously increased the ovulation rates [128]. Clomiphene citrate is associated with an increased risk of multiple pregnancies, despite its effectiveness in improving ovulation [129]. Evidence shows that approximately 14% of female patients develop three or more follicles, despite receiving low doses of clomiphene citrate as treatment [130]. In addition to the increased risk of multiple pregnancies, other common but transient side effects reported for clomiphene citrate include headaches, blurred vision, hot flashes and mood changes [131]. The reported side effects are often mild. Thus, clomiphene citrate remains the first-line therapy for ovulation induction to treat infertility in PCOS patients.

G-15 is an example of a GPER antagonist. It is often used as a manipulating tool to inhibit the expression of GPER during in vitro experiments by examining the effects of GPER inhibition on the changes of important elements for specific biological processes. For instance, G-15 was used in an experiment with the aim of confirming whether inhibition of GPER expression affects insulin secretion and proves the involvement of GPER in insulin regulation [132]. Another study with the aim of studying the involvement of GPER in calcium ion regulation also utilised G-15 as a GPER-manipulating tool [133]. G-15 exhibits excellent antagonistic activity on GPER because it can effectively inhibit GPER expression in various cell types, such as rat myometrial cells [133] and mouse insulinoma MIN6 cells. A recent study featured the use of G-15 for treatment of non-small cell lung cancer induced by oestrogen through GPER activation. Results of the study demonstrated that G-15 successfully decreases the expression of GPER protein in the A549 and H1793 carcinoma cell lines [55]. Based on its effectiveness in inhibiting GPER, G-15 might serve as a suitable candidate for treatment of PCOS-associated conditions caused by overexpressions of GPER, such as abnormal folliculogenesis and endometriosis. 

Vilaprisan is an example of a PR antagonist with a strong binding affinity to PR. It is a common therapeutic option for uterine fibroids [134]. Heavy menstrual bleeding and pelvic pain are two of the most common symptoms of uterine fibroids. Studies have proven vilaprisan to be effective in suppressing menstrual bleeding and pelvic pain in uterine fibroids [135,136]. Moreover, vilaprisan treatment significantly decreased mean average oestradiol and mean maximum progesterone concentrations compared to pretreatment [136]. Vilaprisan was selected as a potential treatment for endometriosis due to its effectiveness in suppressing pelvic pain. An ongoing phase II clinical trial is being conducted to evaluate the efficacy and safety of doses (2 and 4 mg for 24 weeks) of vilaprisan in treating women with symptomatic endometriosis. This research, which measures the changes in the worst pelvic pain from the baseline as the primary outcome, is expected to be completed on 17 August 2022 [137]. However, several adverse events are associated with vilaprisan treatment, including ovulation inhibition, increased follicle size, headache, endometrial disorders (inhomogeneous/cystic endometrium), ovarian cysts, cervical cancer cysts, haemorrhagic ovarian cysts, proteinuria, nausea, hot flashes and dizziness. Most of the side effects are mild, and normal ovulatory cycles resume immediately after vilaprisan treatment; however, ovulation inhibition remains a major problem associated with this treatment [138]. Therefore, an ovulation-stimulating drug may be used as an adjuvant for vilaprisan in PCOS treatment, especially for patients who desire to conceive.

Flutamide is a common AR antagonist used to treat conditions associated with PCOS, such as hirsutism, anovulation and hyperlipidaemia. Flutamide is the most widely used and reported AR antagonist. This drug has a favourable effect in reducing hirsutism and acne amongst PCOS patients [139]. PCOS patients also reported improved menstrual cycle regularity and ovulation following flutamide treatment. These patients recorded decreased luteinising hormone and increased oestrone, as well as oestradiol and 17-hydroxyprogesterone levels [140]. Another study stated that flutamide treatment improved obesity outcomes in female patients with PCOS lipid profiles. The results showed a significant decrease in total cholesterol, LDL and triglycerides amongst obese PCOS patients [141]. However, the use of flutamide can lead to hepatotoxicity, despite its effectiveness in relieving PCOS-associated conditions. This phenomenon represents a major setback in the application of PCOS treatment.

## 5. Limitations

Several limitations of this review are worth highlighting. One limitation is the inclusion of additional in vitro and in vivo studies, which mainly include results on cell lines and animals. In addition, clinical trials involving human subjects included in this review are limited due to ethical restrictions, especially when isolating specimens (i.e., GCs of small antral follicles from normal women to analyse the expression level of sex hormone receptors). Therefore, animals (such as mice and rats) or cell lines (such as endometrial cells) are the study subject for most research involving reproduction and sex hormone receptors. Despite the convenience and cost-saving aspect of using small animal models, the physiological differences between animals and humans may not accurately represent the exact biological response experienced in humans. For example, only the PR-A isoform was necessary for establishment of pregnancy in a murine model, whereas PR-A and PR-B isoforms were equally crucial for human pregnancy [142,143]. Therefore, studying pathophysiological processes involving complicated signalling pathways, such as PCOS, which affects the regulation of different sex hormone receptors, is challenging using only animal models and cell lines.

Many knowledge gaps still exist with respect to sex hormone receptors due to the difficulties in obtaining a suitable study subject. For instance, the changes and interactions between PR-A and PR-B in metabolic regulation during pregnancy are still not fully understood, despite research conducted over a long period. The interaction between AR and PR in mediating endometrial effects in endometriosis pathogenesis remains unclear due to contradictory findings in different publications [144,145]. Furthermore, the therapeutic approach using receptor agonists/antagonists has been proven effective in relieving some of the symptoms of PCOS. However, the appearance of side effects following treatment indicates that a considerable amount of information regarding the receptors should be explored through extensive research.

We realise that the nature of our review as a narrative review that does not include a systematic methodology for choosing articles may have caused us to overlook relevant studies. Future research could adopt a methodical strategy for manuscript selection to enhance our findings.

## 6. Recommendations

The interaction between the three sex hormone receptors, namely ER, PR and AR, which regulate metabolic homeostasis, pregnancy establishment and menstrual cycle, respectively, is complicated. In addition to extensive research, a suitable experimental model should be used to study their complex interactions. Animal models that share common physiological features with humans, such as primates, should be frequently used to ensure proximity to actual human physiological responses. This condition allows for remarkably accurate discovery regarding PCOS pathogenesis and other conditions regulated by the sex hormone receptors, especially PR- and AR-mediated regulations, which are less well-studied than ER-mediated regulation. For instance, extensive research should be conducted to uncover the role of PR-A and PR-B in metabolic regulation during pregnancy. Furthermore, detailed structures and conformations of receptors and their agonists or antagonists should be studied because these pieces of information are essential for manipulating the receptors and their respective agonists/antagonists. By interfering with the structures and conformation of the receptors and their agonists/antagonists, side effects associated with treatments of receptor agonists/antagonists could be eradicated while simultaneously ensuring their potency. The safety and efficacy of the receptor agonists and antagonists should be tested thoroughly in the short and long term to confirm that they are safe to be used as therapeutic agents.

## 7. Conclusions

PCOS and endometriosis are medical conditions associated with infertility in women. Abnormal folliculogenesis and metabolic disorders, including obesity, diabetes mellitus, cardiovascular disease and hypertension, are phenotypes associated with PCOS. These PCOS phenotypes, as well as endometriosis, are caused by dysregulation of sex hormone receptors, namely ER, PR and AR. Therefore, these receptors could be potential targets for PCOS and endometriosis treatments. The expression of ER, PR and AR can be regulated by their respective agonists or antagonists. Hence, receptor agonists and antagonists may serve as potential therapeutic routes for the management of PCOS and endometriosis.

## Data Availability

Not applicable.
